# Ascorbic Acid Changes Growth of Food-Borne Pathogens in the Early Stage of Biofilm Formation

**DOI:** 10.3390/microorganisms8040553

**Published:** 2020-04-11

**Authors:** Jana Przekwas, Natalia Wiktorczyk, Anna Budzyńska, Ewa Wałecka-Zacharska, Eugenia Gospodarek-Komkowska

**Affiliations:** 1 Department of Microbiology, Nicolaus Copernicus University in Toruń, Ludwik Rydygier Collegium Medicum, 9 Maria Skłodowska-Curie Street, 85-094 Bydgoszcz, Poland; natalia12127@gmail.com (N.W.); a.budzynska@cm.umk.pl (A.B.); gospodareke@cm.umk.pl (E.G.-K.); 2 Department of Food Hygiene and Consumer Health, Wrocław University of Environmental and Life Sciences, 31 C.K. Norwida St., 50-375 Wrocław, Poland; ewa.walecka@upwr.edu.pl

**Keywords:** vitamin C, ascorbic acid, biofilm, food, *Listeria monocytogenes*, *Escherichia coli*, *Staphylococcus aureus*

## Abstract

Since bacterial biofilm may contribute to the secondary contamination of food during the manufacturing/processing stage there is a need for new methods allowing its effective eradication. Application of food additives such as vitamin C already used in food industry as antioxidant food industry antioxidants may be a promising solution. The aim of this research was evaluation of the impact of vitamin C (ascorbic acid), in a range of concentrations 2.50 µg mL^−1^–25.0 mg mL^−1^, on biofilms of *Staphylococcus aureus*, *Escherichia coli*, and *Listeria monocytogenes* strains isolated from food. The efficacy of ascorbic acid was assessed based on the reduction of optical density (*λ* = 595 nm). The greatest elimination of the biofilm was achieved at the concentration of vitamin C of 25.0 mg mL^−1^. The effect of the vitamin C on biofilm, however, was strain dependent. The concentration of 25.0 mg mL^−1^ reduced 93.4%, 74.9%, and 40.5% of *E. coli*, *L. monocytogenes*, and *S. aureus* number, respectively. For *E. coli* and *S. aureus* lower concentrations were ineffective. In turn, for *L. monocytogenes* the biofilm inhibition was observed even at the concentration of 0.25 mg mL^−1^. The addition of vitamin C may be helpful in the elimination of bacterial biofilms. Nonetheless, some concentrations can induce growth of the pathogens, posing risk for the consumers’ health.

## 1. Introduction

Foodborne diseases are a serious problem for public health. Their number has been increasing for many years. In the developed countries the proportion of infected people reaches up to 30% every year [1]. In 2010 foodborne diseases resulted in circa 420,000 deaths worldwide, with children under 5 years accounting for 40% of them [2]. According to the EFSA (European and Food Safety Agency) report, since over a decade, the most prevalent diseases in the European Union have been *campylobacteriosis* and *salmonellosis* [3]. In turn, listeriosis and *Escherichia coli* O157:H7 infections are mainly associated with the consumption of RTE (ready to eat) food [4].

Many foodborne pathogens, including *E. coli*, *Salmonella* spp., *Listeria monocytogenes*, and *Staphylococcus aureus* are able to form a biofilm both on the food and food contact surfaces [5,6,7]. Such ability protects bacteria from adverse conditions and helps them to survive in the food production environment, thereby being a great challenge for the assurance of microbiological safety of fresh products. In the mature biofilm bacteria may tolerate up to 1000 times higher concentrations of antimicrobials than in the planktonic form [8]. The ability to form a biofilm is affected by many factors, including surface type (plastic, metal, etc.) and temperature of the environment. For the majority of pathogens, the temperature of the human body is optimal for the growth and biofilm formation. In the food industry, however, room temperature (25 °C) and lower are applied. *L. monocytogenes* is the bacterium well-known for its ability to adapt to wide range of temperatures [9,10] and form a biofilm even at temperatures as low as 0 °C [11]. The ability of *S. aureus* and *E. coli* to colonize the surfaces and form a biofilm at low temperatures on various surfaces also may contribute to bacterial survival in the food industry environment, increasing the risk of food cross-contamination [12]. Therefore, conventional methods of disinfection in food plants may be insufficient to eliminate pathogens [13,14]. Currently, the efficacy of natural compounds extracted from plants and bacteriocins against biofilms has been studied. These substances are regarded as safe and biodegradable and may penetrate the biofilm structure killing the bacteria [14]. The application of vitamin C is one of such alternatives. Vitamin C is cheap, easily accessible, and has been reported to have antimicrobial activity against *S. aureus, Enterococcus faecalis* [15], *Mycobacterium tuberculosis* [16], and *Aspergillus* spp. [17]. Additionally, vitamin C may augment the inhibiting action of antibiotics i.e., levofloxacin and azithromycin [17]. It is a food additive (E300), applied as an acidity regulator, antioxidant, food improver, and sequestrant in many types of food products e.g., milk, fresh produce, frozen food, pasta, meat, fish, flour, juice. Depending on the product its maximal concentration ranges from 50 to 500 mg/kg. The inhibiting action of ascorbic acid may be associated with its anti-quorum sensing activity as it competes with the autoinducer-2 (AI-2) [18]. It has been also suggested that vitamin C inhibits extracellular polymeric substances production, the major biofilm component, destabilizing the biofilm structure [19].

The new method of food preservation is active antimicrobial packaging. The idea is to use the antimicrobial material as packaging and/or coat the packaging material with antimicrobial agents, such as organic acids, bacteriocins, silver, enzymes, essential oils, and parabens. This solution may affect only the surface of the packaging and the product in contact with packaging or, if the antimicrobial agent is volatile or soluble, it can penetrate to liquid products, such as juices [20,21].

Vitamin C, as organic compound, could be possibly incorporated into packages made from edible films (e.g., polysaccharides, like chitosan or starch, but also proteins (collagen, gelatin)) or lipids (beeswax, paraffin, resins) or polyethylene, e.g., EVA (ethyl vinyl acetate) or LLDPE (linear low density polyethylene) [22].

The aim of this study was to assess the impact of vitamin C on *L. monocytogenes, S. aureus*, and *E. coli* strains in the early stage of biofilm formation. This is a pilot study for the assessment of vitamin C as a possible anti-biofilm agent to incorporate in active antimicrobial packaging materials.

## 2. Materials and Methods

### 2.1. Bacterial Strains

The study was conducted on 18 *L. monocytogenes* strains isolated form frozen vegetables and salmon, 15 *E. coli* strains, and 13 *S. aureus* strains derived from cow milk. Strains were isolated through years 2013–2017. In addition, three reference strains were included: *Listeria monocytogenes* ATCC^®^19111™, *Staphylococcus aureus* ATCC^®^35556™, and *Escherichia coli* ATCC^®^25922™. All strains came from the collection of the Department of Microbiology, Ludwik Rydygier Collegium Medicum in Bydgoszcz, Nicolaus Copernicus University in Toruń, Poland. Strains were deposited in Brain Heart Infusion (BHI, Becton-Dickinson, Franklin Lakes, NJ, USA) broth with 20% of glycerol (Avantor, Gliwice, Poland) at −70 °C.

### 2.2. Biofilm Formation

Before examination, strains were plated on Columbia Agar Base enriched with 5% sheep blood (CAB-SB, bioMérieux, Marcy-l’Étoile, France) using the striking method. Cultures were incubated at 37 °C for 24 h. Obtained single colonies were cultured on CAB-SB (and incubated for next 24 h at 37 °C. Then bacterial suspensions (0.5 McFarland scale) in BHI broth, corresponding to 7.80 × 10^7^ (±1.66 × 10^7^) CFU × cm^−3^ of *L. monocytogenes*, 5.20 × 10^8^ (±2.86 × 10^7^) CFU × cm^−3^ of *E. coli*, and 1.72 × 10^8^ (±4.17 × 10^7^) CFU × cm^−3^ of *S. aureus*, were prepared. Such suspensions were diluted 1:100 (*v/v*) in BHI broth and deposited in 96-well plates (Profilab, Warszawa, Poland) (200 µL). After 24 h of incubation in a humid chamber at 37 °C planktonic cells were removed and wells were washed four times with sterile phosphate buffered saline (PBS, BTL, Łódź, Poland). For *L. monocytogenes* after 24 h the wells were washed, and the medium was replaced with the fresh one for additional 24 h to prevent planktonic cells from multiplication and instead provide the conditions for the biofilm to establish. The negative control was sterile BHI broth. Reference strains were included and treated the same as other strains.

### 2.3. The Assessment of Ascorbic Acid Addition on Bacterial Biofilm Elimination

The ascorbic acid (Sigma-Aldrich, Saint Louis, MI, USA) at five concentrations, 2.50 µg mL^−1^, 25.0 µg mL^−1^, 0.25 mg mL^−1^, 2.50 mg mL^−1^, and 25.0 mg mL^−1^, was added to wells washed with PBS containing bacterial biofilm. After 24-h incubation at 37 °C wells were washed four times with PBS, refilled with PBS and sonicated (Ultrasonic DU-4 sonicator, Nickel-Electro, Oldmixon, Great Britain, 20 min). Finally, optical density (*λ* = 595 nm) was measured (Bio-Tek, Synergy HT, Winooski, VT, USA) [23]. For each strain the positive control was bacterial biofilms treated with sterile BHI (not exposed to vitamin C treatment). The reduction of optical density was calculated according to the formula:

Reduction (%) = [(OD _K(+)_ − OD)/OD _K(+)_] × 100% (1)

where:

OD _K(+)_—optical density of the positive control;
OD—optical density of the biofilm treated with vitamin C.

### 2.4. Statistical Analysis

Each experiment was repeated three times. Statistical analysis was performed in Statistica 13 (TIBCO Software, Palo Alto, CA, USA). To test whether significant differences exist between different experimental groups one-way ANOVA with the Tukey post-hoc test and the sign test were used. A *p* value above 0.05 was considered statistically significant.

Strains of each species were also divided into two groups: weak and strong biofilm producers based on cut-off point (OD_c_) and positive control (OD_K(+)_) comparison (Table 1).

OD_c_ is defined as optical density cut-off value and calculated according to Equation (2):

OD_c_ = x + (3 × SD) (2)

where:

OD_c_—optical density cut-off value;
x—average optical density of negative control;
SD—standard deviation of optical density of negative control.

Groups of weak and strong biofilm producers were compared using one-way ANOVA with the Tukey post-hoc test. A *p* value above 0.05 was considered statistically significant.

## 3. Results

**Figure 1:**
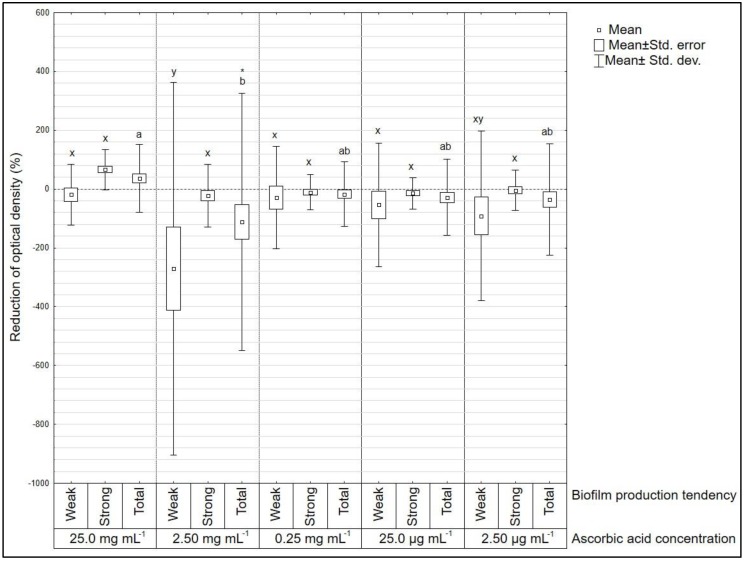
Reduction of optical density (%) after ascorbic acid treatment on *Staphylococcus aureus* (*n* = 14) strains. Amongst all strains, *n* = 5 were defined as weak biofilm producers and *n* = 9 as strong biofilm producers. Letters a, b mark statistically significant differences between different concentrations (all strains included). Letters x, y mark statistically significant differences between groups of strong and weak biofilm producers (*p* < 0.05). * Statistically significant difference between certain ascorbic acid concentration and positive control (marked with horizontal line, value “0” on y axis).

From the tested concentrations of vitamin C, 25.0 mg mL^−1^ most efficiently inhibited bacterial growth in the biofilm. The average inhibited bacterial growth for *E coli*, *L. monocytogenes*, and *S. aureus* were 93.4%, 74.9%, and 40.5%, respectively. The sensitivity to vitamin C, however, was strain dependent. For *E. coli*, only the concentration of 25.0 mg mL^−1^ of vitamin C significantly reduced the optical density, whereas for *L. monocytogenes* the inhibition was observed at concentrations ranging from 0.25 to 25.0 mg mL^−1^. For *S. aureus* no concentration significantly reduced optical density, in fact, the concentration of 2.50 mg mL^−1^ significantly increased optical density (Figure 1). The concentration of 2.50 mg mL^−1^ increased optical density of 115.9%. ANOVA analysis and post-hoc Tukey test confirmed that for *S. aureus* there was a statistically significant difference between concentrations 25.0 and 2.50 mg mL^−1^. The comparison of weak and strong biofilm producers of *S. aureus* showed that there was no statistically significant difference between these groups, with the exception of the concentration of 2.50 mg mL^−1^.

**Figure 2:**
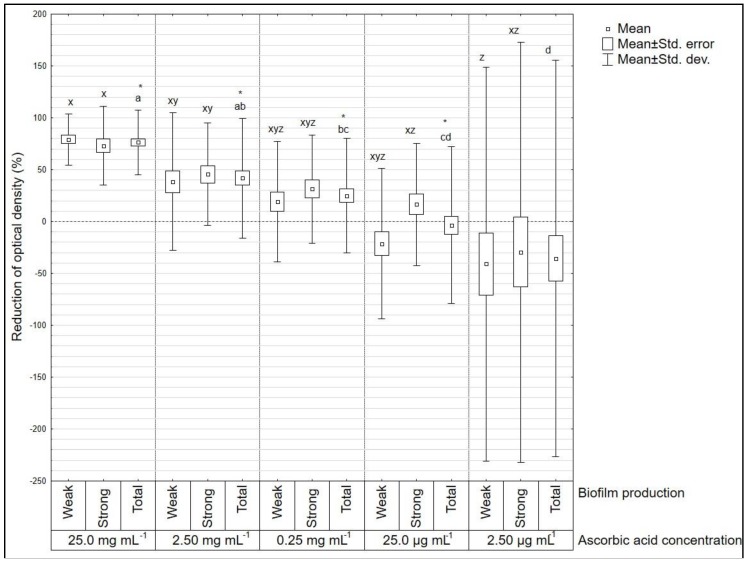
Reduction of optical density (%) after ascorbic acid treatment on *Listeria monocytogenes* (*n* = 19) strains. Amongst all strains *n* = 10 were defined as weak biofilm producers and *n* = 9 as strong biofilm producers. Letters a, b, c, d mark statistically significant differences between different concentrations (all strains included). Letters x, y, z mark statistically significant differences between groups of strong and weak biofilm producers (*p* < 0.05). * Statistically significant difference between certain ascorbic acid concentration and positive control (marked with horizontal line, value “0” on y axis).

For *L. monocytogenes* (Figure 2) statistically significant differences after vitamin C treatment compared to the positive control were noted for the concentrations of 25.0 mg mL^−1^, 2.50 mg mL^−1^, 0.25 mg mL^−1^, and 25.0 µg mL^−1^. Only for the concentration of 2.50 µg mL^−1^ no difference compared to the positive control was observed. There were no statistically significant differences between strong and weak biofilm producer groups.

**Figure 3:**
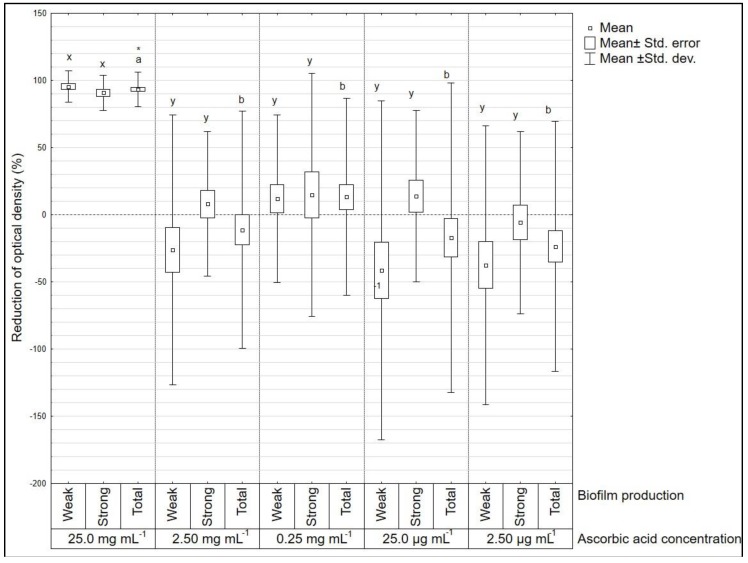
Reduction of optical density (%) after ascorbic acid treatment on *Escherichia coli* (*n* = 16) strains. Amongst all strains *n* = 9 were defined as weak biofilm producers and *n* = 7 as strong biofilm producers. Letters a, b mark statistically significant differences between different concentrations (all strains included). Letters x, y mark statistically significant differences between groups of strong and weak biofilm producers (*p* < 0.05). * Statistically significant difference between certain ascorbic acid concentration and positive control (marked with horizontal line, value “0” on y axis).

For *E. coli* sign test (Figure 3) a statistically significant difference between the treated group and the positive control was only found for the concentration of 25.0 mg mL^−1^. This was confirmed by the one-way ANOVA analysis and the Tukey post-hoc test. No statistically significant differences between strong and weak biofilm former groups were found.

## 4. Discussion

Bacterial biofilms are a serious problem in the food industry. In such a structure, pathogenic bacteria may survive for a long time and contribute to the secondary contamination of food products, thereby posing a risk for the consumer. Therefore, it is of great importance to eliminate biofilms from the food production environment. One method is the application of safe and biodegradable food additives as antimicrobial agents in active antimicrobial packaging. Vitamin C is commonly applied in the food industry and has been shown to inhibit bacterial growth. Since this substance is easily accessible and cheap it may be an alternative method of pathogen elimination. To date, many studies have reported its antibacterial activity against *S. aureus, E. faecalis Helicobacter pylori, L. monocytogenes*, *Campylobacter jejuni*, *M. tuberculosis*, and *Aspergillus* spp. [15,16,17,24,25].

Several studies have also reported the inhibitive or stimulative impact of ascorbic acid on biofilm formation, established biofilm and colony spreading of *S. aureus*, *Pseudomonas aeruginosa,* and *Bacillus subtilis*. [26,27,28]. However, still little is known about the effect of different concentrations of vitamin C on foodborne pathogens in the early stage of biofilm formation.

In the present study the role of ascorbic acid on biofilms of *S. aureus*, *E. coli*, and *L. monocytogenes* was evaluated. We found the best antimicrobial activity of vitamin C in the concentration of 25.0 mg mL^−1^. The sensitivity to vitamin C was species dependent. The greatest biofilm growth inhibition was noted for *E. coli*, whereas *S. aureus* was the least susceptible. For *E. coli* inhibition was observed only for the highest concentration applied (25.0 mg mL^−1^). Moreover, the concentration of 2.50 mg mL^−1^ stimulated the growth of *S. aureus*. On the other hand, in the case of *L. monocytogenes* the optical density reduction was achieved also at lower concentrations (2.50 and 0.25 mg mL^−1^). In this study no correlation between the strength of biofilm formation ability and the sensitivity to vitamin C treatment was observed. Tabak et al. [29] have also found that ascorbic acid in concentrations of 0.2% to 2.0% inhibited the growth of *H. pylori* in liquid medium. Isela et al. [15] have shown that MIC (Minimum Inhibitory Concentration) of vitamin C for *S*. *aureus* was 10 mg mL^−1^ and the concentration of 20 mg mL^−1^ reduced 90.0% of bacteria [15]. In turn, Mirani et al. [26] have observed that ascorbic acid inhibited EPS production and *staphylococcal* biofilm formation but the survived bacteria tolerated its toxic concentration. Helgadóttir et al. [30] have noticed the reduction of 89.9% of *S. epidermidis, E. coli*, and *P. aeruginosa* bacteria number on catheters after vitamin C treatment. Similar results against mature biofilms of uropathogenic bacteria (*E. coli, Klebsiella* spp., *Citrobacter* spp., *Enterobacter* spp., *Proteus* spp., and *Pseudomonas* spp.) have been reported by El-Gebaly et al. [31]. On the contrary, other studies [30,32,33,34] have found that ascorbic acid did not inhibit the growth of *E. coli* and *P. aeruginosa* but made them more resistant to antimicrobials and physical disinfection.

In the present study biofilm formation was examined at 37 °C, as it is the optimal temperature for the vast majority of human pathogens. However, for the idea of vitamin C application as an anti-biofilm agent in the food industry lower temperatures may play also important role. Air temperature has an impact on cell surface properties, movement ability, and virulence factors expression [35]. For example, *L. monocytogenes* can grow and form a biofilm at wide range of temperatures, even as low as 4 °C [36]. Synthesis of the cilia, an element crucial in biofilm formation by *L. monocytogenes*, is temperature-dependent (20–25 °C) [37]. About 20% of *L. monocytogenes* clinical isolates has the ability to form cilia at 37 °C [38]. Piercey et al. [39] have shown that *L. monocytogenes* isolates formed biofilm more effectively at a temperature of 30 °C than at 37 °C. The main tendency, however, is the weakening of biofilm production with a temperature decrease [39,40]. The ability of *S. aureus* to adhere to and to form a biofilm on surfaces such as polystyrene, polypropylene, and stainless steel is well-known [41,42,43]. Silva Meira et al. [43] documented that the temperature of incubation (7 and 28 °C) has no significant effect on biofilm formation by *S. aureus* on stainless steel and polypropylene. Pagedor et al. [42] have shown a higher number of *S. aureus* biofilm cells at 25 °C compared to 37 °C on stainless steel. Di Ciccio et al. [41] have found that only one of 67 *S. aureus* strains isolated from food was able to form a biofilm on polystyrene and stainless steel at 12 °C. Temperature is also a cue for the gene expression regulation in *E. coli.* White-Ziegler et al. [44] have shown the increased expression of the cilia genes in *E. coli* and concluded that low temperature is a significant environmental cue used to enhance the expression of a few biofilm genes in *E. coli*.

The presented study is a pilot investigation aiming to assess the effect of vitamin C on bacteria at the first stage of biofilm formation. Nonetheless, further research is needed to evaluate the influence of lower temperatures, used in the food industry, as well as the stage of biofilm formation on the antibacterial efficacy of ascorbic acid.

Based on the current research and previous studies it can be assumed that the application of vitamin C in the food industry can be an alternative method of the inhibition of bacterial growth and biofilm eradication. Its mechanism of action includes not only anti-quorum sensing activity [18] and inhibition of extracellular polymeric substances production [19], but also its ability to lower the pH in the environment, providing unsuitable conditions for bacteria to survive.

Vitamin C is more often used in active packages as an antioxidant agent [45,46]. Lee J-S. et al. [46] have demonstrated higher oxygen scavenging properties of activated carbon-ascorbic acid active packaging than standard iron powder. In addition, they have shown that ascorbic acid active packaging significantly inhibited growth of aerobic bacteria, yeasts, and molds on meatloaves during 4 days of incubation at 4 °C [46].

Nevertheless, vitamin C efficacy is species dependent and is strongly correlated with the used concentration. A dose too low may exert a reverse effect and stimulate the bacterial growth. In turn, a dose too high may impact the organoleptic properties of food and consumer health. According to the Regulation (EC) No 13333/2008 of the European Parliament [47] the maximal dose of vitamin C in the food ranges from 50 to 500 mg/kg, depending on the product type. Stepien et al. [48] have suggested that extremely high intake of this vitamin may lead to kidney stones and acute kidney failure. Nonetheless, Curhan et al. [49] have not shown the relations between high daily intake of vitamin C and kidney stones. The others have stated that side effects are rare and occur at doses over 4 g per day [50].

## 5. Conclusions

Currently, researchers have been looking for antibiofilm agents that are based on natural compounds and are easily biodegradable. Vitamin C, commonly used as a food additive, seems to be a good alternative. We showed that ascorbic acid, in the appropriate concentration, reduces bacterial growth in the biofilm of *S. aureus*, *L. monocytogenes*, and *E. coli*. However, further studies are needed to establish a dose that will be effective for the most prevalent foodborne pathogens, safe for consumers, and will not influence food quality.

## Figures and Tables

**Table 1:** Cut-off point for differentiation of weak and strong biofilm producers.

OD _K(+)_/OD_c_	Biofilm Production
≤3	Weak
>3	Strong
